# The Comradeship of Science

**Published:** 1924-09

**Authors:** 


### THE COMRADESHIP OF SCIENCE.

### WORK OF THE ROCKEFELLER FOUNDATION.

"Science as a common fund to which all nations contribute and from which each may freely draw grows steadily in volume and in value. The world is dotted with centres of research and with individuals who are in quest of truth. We can trace the outlines at least of a vast co-operation which tends more and more to ignore national frontiers. In this team work of the nations the medical scientists and the sanitarians have an inspiring part. They not only feel the thrill of discovery and of high adventure in coping with the problems which challenge their knowledge and skill, but they know the satisfaction of safeguarding life and of alleviating suffering." It is in this way that Mr. George E. Vincent, the President of the Rockefeller Foundation, writes in his review for 1923. It is in this spirit of comradeship that the vast resources of the Foundation are administered. The work is performed largely through departmental organisations that are devoted to special functions and the income administered reaches the huge total of 8½ million dollars. Dr. Frederick J. Russell succeeded Mr. Wickliffe Rose in 1923 as the Director of the International Health Board, which is the principal of the departmental organisations.

### "The Peaceful Strife of Science."

The phrase is Pasteur's. In the recruiting and training of young scientists by promoting international migration, the Foundation provides for the entry into the pacific field of science of men who will strive for the pre-eminence of their severa countries. Either directly or through other agencies it provided in 1923 fellowships for 636 men and women who were preparing for teaching or administration in public health, medicine, biology, physics, chemistry, medical and premedical education, and nursing. It is not enough that many young workers should get a part of their training in foreign lands. Older persons, already in important official positions, need experience abroad, the chance to make comparisons, to get new ideas. It was a happy idea of the Health Section of the League of Nations to establish what are termed interchanges of health officials. The value of such meetings as these is unquestionable. The International Health Board is providing the money for a period of years to meet the expenses of these interchanges.

### Medical Work in the Far East.

The visitor to Peking to-day who has had no warning in advance is surprised to find on the site of what was once the palace of a Chinese prince a group of beautiful buildings which at first glance seem to be of classic Chinese architecture. But on closer examination other features are noted. Here evidently is an institution of the West which has assumed some outer aspects of the East. It is the Peking Union Medical College, built, equipped, and maintained with funds supplied by the Rockefeller Foundation through the China Medical Board. In these laboratories, classrooms, and hospital pavilions teaching and research are being carried on :—

Soon after the Japanese disaster it was possible to send promptly to Tokyo from the Peking Union Medical College not only a shipment of foodstuffs and clothing but certain equipment and supplies. Later the hospitality of the College laboratories was offered to the Japanese Government, which sent a group of eight investigators who are remaining a number of months as guests of the College.

### Safeguarding the German Research Worker.

The plight of young medical scientists in the Central European countries and the Balkans has recently become so critical that the continuity of workers has been seriously threatened. In Germany especially the danger of a breakdown has aroused the anxiety of the scientific world :—

The Rockefeller Foundation, in the interest primarily of modern medicine, therefore asked a committee of German scientists to select promising younger workers who, if they had no aid, would be compelled to turn to other pursuits, and to appoint them to "resident fellowships." These provide small stipends together with sums for laboratory supplies and experimental animals. In 1923 the committee granted 194 of these fellowships. The trustees have authorised the extension of this plan to other countries in which similar conditions may be found.

### Many Fields of Activity.

Our space permits only of a mere reference to the fact that the Foundation has provided large sums for helping Brazil in her final bout with yellow fever, for lending a hand in many quarters with nurse training, in the hookworm and malaria campaigns, and for medical education generally, and especially in the teaching of public health. It will be remembered that the new Anatomy building for the University College Hospital Medical School, opened in 1923 by the King, was erected by Rockefeller funds. A recent article in this Review gave an account of the munificence of the Foundation in providing funds for a London School of Hygiene and Tropical Medicine. Large sums for similar purposes are being spent in America and elsewhere in Europe :—

The International Health Board has endowed a School of Hygiene and Public Health at Johns Hopkins University, has enabled Harvard University to reorganise its health courses into a new School of Public Health, has agreed to provide land, buildings, and equipment for a School of Hygiene and Tropical Medicine in London, and has contributed substantially to institutes of public health in Prague and Warsaw.

### World-Wide Help, With Limitations.

It is right that we should record some of the definite limitations which the Foundation finds it necessary to place on its beneficent activities. The Foundation consistently declines to make gifts or loans to individuals, to contribute to the building or maintenance of churches, hospitals (except in connection with educational programmes), and other local institutions, or to support campaigns to influence public opinion on social or political questions. But by fostering medical science and public health as forms of international co-operation, the Rockefeller Foundation seeks to fulfil the purpose of its charter, "the well-being of mankind throughout the world."

